# Twisted states in nonlocally coupled phase oscillators with frequency distribution consisting of two Lorentzian distributions with the same mean frequency and different widths

**DOI:** 10.1371/journal.pone.0213471

**Published:** 2019-03-12

**Authors:** Yuan Xie, Lan Zhang, Shuangjian Guo, Qionglin Dai, Junzhong Yang

**Affiliations:** School of Science, Beijing University of Posts and Telecommunications, Beijing, China; Technical University of Madrid, SPAIN

## Abstract

In globally coupled phase oscillators, the distribution of natural frequency has strong effects on both synchronization transition and synchronous dynamics. In this work, we study a ring of nonlocally coupled phase oscillators with the frequency distribution made up of two Lorentzians with the same center frequency but with different half widths. Using the Ott-Antonsen ansatz, we derive a reduced model in the continuum limit. Based on the reduced model, we analyze the stability of the incoherent state and find the existence of multiple stability islands for the incoherent state depending on the parameters. Furthermore, we numerically simulate the reduced model and find a large number of twisted states resulting from the instabilities of the incoherent state with respect to different spatial modes. For some winding numbers, the stability region of the corresponding twisted state consists of two disjoint parameter regions, one for the intermediate coupling strength and the other for the strong coupling strength.

## Introduction

It is well known that natural frequency distribution *g*(*ω*) plays a critical role in displaying rich synchronous dynamics in globally coupled phase oscillators. For unimodal frequency distribution, the partial synchronous state steps in through a continuous transition when the coupling strength is above a critical coupling strength [1]. Further increasing the coupling strength, the partial synchronous state may transit to a global synchronization where all oscillators oscillate at a same frequency. For a bimodal frequency distribution, increasing coupling strength always first leads to a standing wave state and then to a traveling wave state [2]. When the peak distance in the bimodal frequency distribution is narrow, the discontinuous transitions between different dynamical states are possible. When the frequency distribution becomes more complicated, for example a trimodal one, the synchronous dynamics in globally coupled phase oscillators may display collective chaos through a cascade of period-doubling bifurcations [3]. In the above works, incoherent state is always unstable when the coupling strength is above a threshold and further increasing coupling strength always enhances synchronization among oscillators. Recently, some authors studied the synchronous dynamics when the frequency distribution is a superposition of two unimodal frequency distributions with the same mean frequency in a globally coupled oscillator system [4]. They found a non-universal synchronization transition in which the incoherent state may be restored for the coupling strength above the threshold.

The impacts of the frequency distribution on the synchronous dynamics possibly exist in other types of coupling topologies, for example in nonlocally coupled phase oscillators. A system of nonlocally coupled phase oscillators has become an important platform for investigating collective dynamics among oscillators. One interesting dynamics is the chimera state in which synchronous clusters and desynchronous clusters coexist [5–12]. Twisted state is another interesting type of collective dynamics and is a simpler one [13–17]. Twisted state was first identified in a ring of coupled identical phase oscillators and is characterized by the linear dependence of the phase of oscillator on oscillator’s spatial position. A twisted state with winding number *q* refers to a state in which the phase of oscillator increases linearly along the ring and varies 2*πq* through the ring. Wiley et al. found coexistence of a large number of twisted states in nonlocally coupled identical phase oscillators and the probability finding a twisted state with winding number *q* follows a Gaussian distribution with respect to *q* [13]. Lately, the investigation on twisted states was generalized to non-identical phase oscillators with unimodal frequency distribution and the concept of partially coherent twisted states was proposed [15]. Beyond a critical coupling strength, the incoherent state becomes unstable and partially coherent twisted states step in. In partially coherent twisted states, the existence of asynchronous oscillators breaks the linear dependence of oscillator’s phase on position. Nevertheless, by introducing a spatially-dependent complex order parameter, it can be found that the argument of the order parameter varies 2*qπ* through the ring in a partially coherent twisted state. Recently, we studied nonlocally coupled phase oscillators with bimodal frequency distribution and found twisted standing waves besides twisted traveling waves [17]. In these works on twisted states, incoherent state remains unstable once twisted states appear and the coherence among oscillators, characterized by the amplitude of spatially-dependent order parameter, always increases with the coupling strength. Now, it is interesting to ask whether the superposition of two unimodal frequency distributions may dispute these observations and how it influences the synchronous dynamics in nonlocally coupled phase oscillators.

In this work, we investigate the effects of the superposition of two unimodal frequency distributions on the incoherent state and twisted states in a ring of nonlocally coupled phase oscillators. We find that, under proper parameters, increasing the coupling strength may encounter multiple stability islands of the incoherent state. We also find that, for some winding numbers, the stability region of the corresponding twisted state consists of two disjoint parameter regions, one for the intermediate coupling strength and the other for the strong coupling strength, and all of these twisted states are related to the instabilities of the incoherent state with respect to different spatial modes. Moreover, we discover the existence of complicated spatiotemporal patterns which have not been observed in previous works mentioned above.

The paper is organized as follows. Firstly, we will describe the model and derive a reduced model in the continuum limit by using the Ott-Antonsen (OA) ansatz. Then, we theoretically investigate the stability of the incoherent state based on the reduced model and we present numerical results on different types of twisted states. The connection between different twisted states and the instabilities of the incoherent state with respect to different spatial modes is proposed. Finally, we will present a brief summary.

## Materials and methods

We consider *N* phase oscillators which sit evenly on a ring with the length 2*π* and nonlocally interact with each other with the coupling strength *K*. The model is described as

$$\begin{array}{c} {\dot{\theta }}_{j}=\omega_{j}+\frac{K}{2M+1}\sum_{k=-M}^{k=M}\sin\left(\theta_{j+k}-\theta_{j}+\alpha \right). \end{array}$$ (1)

The subscript *j* denotes the unit index, which has to be taken modulo *N*. *M* denotes the coupling range and, in the following, we use the coupling radius *σ* = *M*/*N* as a controlling parameter and 0 < *σ* < 0.5. The phase lag *α* is in the range of (0, *π*/2). *ω*_*j*_ is the natural frequency of oscillator *j* located at *x*_*j*_ = 2*πj*/*N*, which follows the frequency distribution *g*(*ω*). In this work, we let *g*(*ω*) be a superposition of two Lorentzian distributions

$$\begin{array}{c} g\left(\omega \right)=p\frac{\gamma_{1}}{\pi }\frac{1}{\omega^{2}+\gamma_{1}^{2}}+\left(1-p\right)\frac{\gamma_{2}}{\pi }\frac{1}{\omega^{2}+\gamma_{2}^{2}} \end{array}$$ (2)

with *p* in the range of [0, 1]. *γ*_1,2_ are the half widths of the two Lorentzians. When *p* = 0 or *p* = 1, the frequency distribution reduces to a normal Lorentzian one. Unless specified, *γ*_1_ = 1, *γ*_2_ = 0.01, and *p* = 0.9.

Partially coherent twisted states can be characterized by the spatially-dependent complex order parameter $Z_{j}=R_{j}e^{i\Theta_{j}}$ which is defined as

$$\begin{array}{ccc} Z_{j} & = & \frac{1}{2M+1}\sum_{k=-M}^{k=M}e^{i\theta_{j+k}}. \end{array}$$ (3)

When *R*_*j*_ becomes nonzero, partially coherent states step in. On the other hand, the dependence of Θ_*j*_ on oscillator’s position distinguishes different twisted states.

It has been shown in Refs. [15, 17] that partially coherent twisted states and their stabilities can be theoretically investigated in the continuum limit based on the OA ansatz [18, 19]. In the limit *N* → ∞, we introduce the probability density function *f* such that *f*(*ω*, *x*, *θ*, *t*)*dθdω* is the fraction of oscillators with phases between *θ* and *θ* + *dθ* and natural frequencies between *ω* and *ω* + *dω* at time *t* and position *x*. The spatially-dependent complex order parameter *Z*(*x*, *t*) is reformulated as

$$\begin{array}{c} Z\left(x,t\right)=\int_{0}^{2\pi }G\left(x-y\right)\int_{-\infty }^{\infty }\int_{0}^{2\pi }fe^{i\theta }d\theta d\omega dy \end{array}$$ (4)

with

$$\begin{array}{c} G\left(x\right)=\{\begin{array}{ccc} \frac{1}{4\pi \sigma }, & & |x|<2\pi \sigma \\ 0, & & \text{otherwise}. \end{array} \end{array}$$ (5)

The evolution of the density *f*(*ω*, *x*, *θ*, *t*) obeys the continuity equation

$$\begin{array}{c} \frac{\partial f}{\partial t}+\frac{\partial }{\partial \theta }\left\{f\left[\omega +K\text{Im}\left(Ze^{-i(\theta -\alpha )}\right)\right]\right\}=0. \end{array}$$ (6)

Using the OA ansatz [18, 19], we have

$$\begin{array}{c} f\left(\omega ,x,\theta ,t\right)=\frac{g(\omega )}{2\pi }\left\{1+\sum_{n=1}^{\infty }{\left[\bar{a}\left(\omega ,x,t\right)\right]}^{n}e^{in\theta }+c.c.\right\}, \end{array}$$ (7)

where **c.c.** is the complex conjugate of the previous term. By substituting Eq (7) into Eq (6) and following the procedures in Refs. [15, 17], we have

$$\begin{array}{c} \frac{\partial u_{1}\left(x,t\right)}{\partial t}=-\gamma_{1}u_{1}\left(x,t\right)+\frac{K}{2}\left[Ze^{-i\alpha }-\bar{Z}e^{i\alpha }u_{1}^{2}\left(x,t\right)\right]\\ \frac{\partial u_{2}\left(x,t\right)}{\partial t}=-\gamma_{2}u_{2}\left(x,t\right)+\frac{K}{2}\left[Ze^{-i\alpha }-\bar{Z}e^{i\alpha }u_{2}^{2}\left(x,t\right)\right] \end{array}$$ (8)

with *u*_1,2_(*x*, *t*) = *a*(−*iγ*_1,2_, *x*, *t*) = |*u*_1,2_(*x*, *t*)|*e*^*iϕ*_1,2_(*x*, *t*)^, measuring the coherence in the subpopulations of oscillators with natural frequency following $\frac{\gamma_{1,2}}{\pi }\frac{1}{\omega^{2}+\gamma_{1,2}^{2}}$, and $Z\left(x,t\right)=\int_{0}^{2\pi }G\left(x-y\right)\left[pu_{1}\left(y,t\right)+\left(1-p\right)u_{2}\left(y,t\right)\right]dy$.

## Results and discussion

The dynamics in Eq (1) can be reproduced by Eq (8). Therefore, we study the stability of the incoherent state in the model (1) by linearizing Eq (8) around it and the synchronous dynamics in the model (1) by performing numerical simulations on Eq (8). Traditionally, the coherence in Eqs (1) and (8) is described by the global complex order parameter defined by $Re^{i\Phi }=\int_{0}^{2\pi }\left[pu_{1}\left(y,t\right)+\left(1-p\right)u_{2}\left(y,t\right)\right]dy$. However, *R* cannot provide correct information on twisted states with nonzero *q*. Considering that, in Eq (8), *u*_1,2_(*x*, *t*) is driven by spatially-dependent complex order parameter *Z*(*x*, *t*), we measure the coherence in Eq (8) by another global quantity 〈|*Z*|〉, defined as $\langle |Z|\rangle =\int_{0}^{2\pi }\left|Z\left(x,t\right)\right|dx/2\pi$. 〈|*Z*|〉 ≠ 0 indicates a synchronous state while 〈|*Z*|〉 = 0 the incoherent state.

### Incoherent state

**Fig 1:**
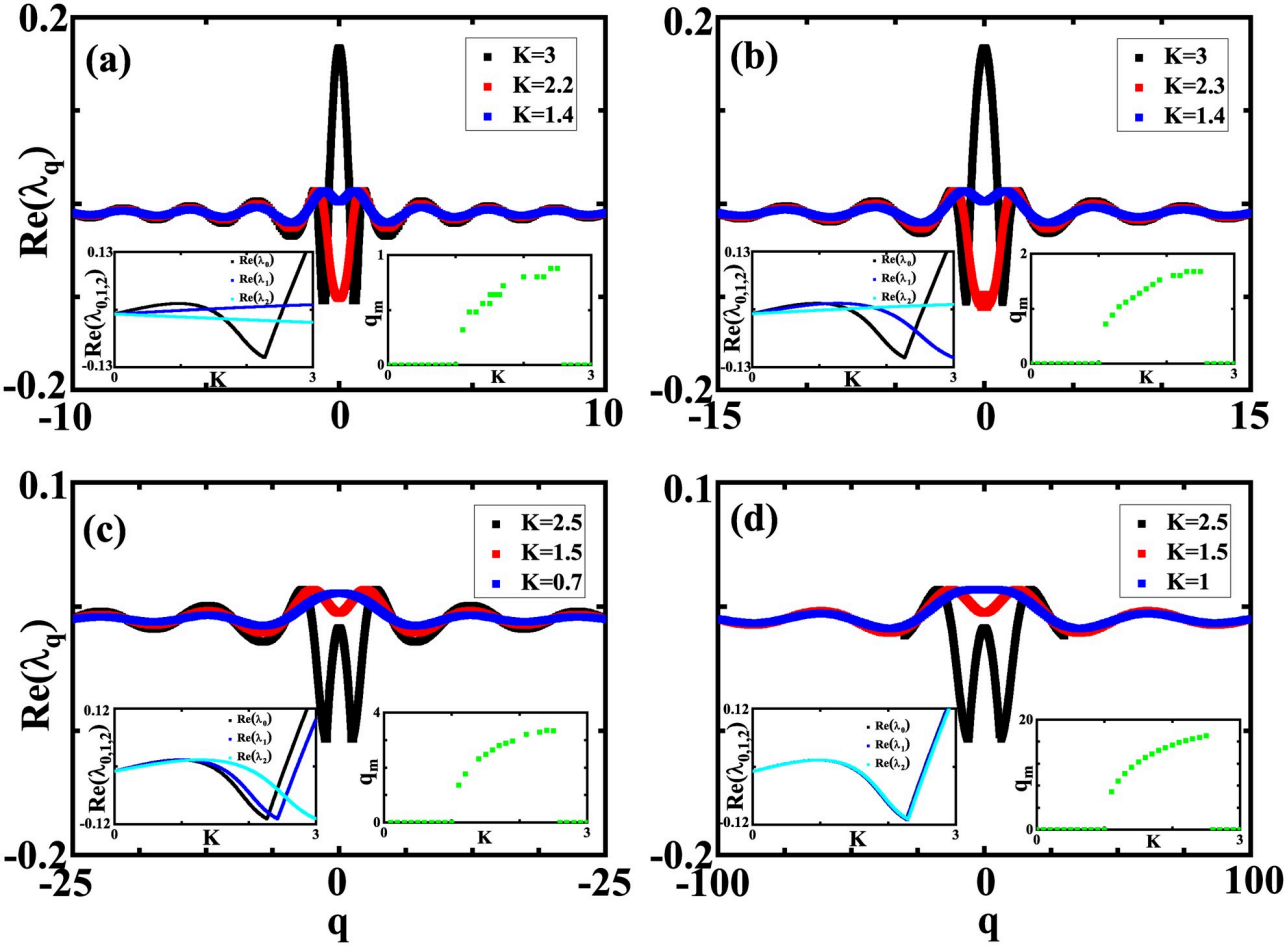
The growth rate λ_*q*_ against *q*. The growth rate λ_*q*_ obtained from Eq (10) is plotted against real number *q* for different coupling strength. The left inset in each panel shows *Re*(λ_0_), *Re*(λ_1_) and *Re*(λ_2_) against *K*, while the right inset shows *q*_*m*_ at which *Re*(λ_*q*_) reaches its maximum against *K*. (a) *σ* = 0.4, (b) *σ* = 0.2, (c) *σ* = 0.1, (d) *σ* = 0.02. *γ*_1_ = 1, *γ*_2_ = 0.01, *α* = 0.8, and *p* = 0.9.

We start with the stability of the incoherent state. The incoherent state is characterized by *u*_1,2_(*x*, *t*) = 0, and hence by *Z*(*x*, *t*) = 0. Linearizing Eq (8) around the incoherent state, we have the evolution of perturbation *δu*_1,2_

$$\begin{array}{c} \frac{d\delta u_{1,2}}{dt}=-\gamma_{1,2}\delta u_{1,2}+\frac{K}{2}e^{-i\alpha }\delta Z \end{array}$$ (9)

with $\delta Z=\int_{0}^{2\pi }G\left(x-y\right)\left[p\delta u_{1}\left(y,t\right)+\left(1-p\right)\delta u_{2}\left(y,t\right)\right]dy$. Supposing that the perturbation takes the form *δu*_1,2_(*x*, *t*) = *v*_1,2_ *e*^*iqx*^ *e*^λ_*q*_*t*^ with the wave number *q* an arbitrary integer (that is, the perturbation on the spatial mode *e*^*iqx*^), we find the spectrum equation

$$\begin{array}{c} 0=\lambda_{q}^{2}+\gamma_{1}\gamma_{2}+\lambda_{q}\left[\gamma_{1}+\gamma_{2}-\frac{K}{2}e^{-i\alpha }\hat{G}\left(q\right)\right]-\frac{K}{2}e^{-i\alpha }\hat{G}\left(q\right)\left[\left(1-p\right)\gamma_{1}+p\gamma_{2}\right], \end{array}$$ (10)

where $\hat{G}\left(q\right)=\sin\left(2\pi \sigma q\right)/2\pi \sigma q$ is the Fourier transform of the coupling function *G*. For any *q*, there are two solutions to Eq (10). The one with the larger *Re*(λ_*q*_), termed as the growth rate of the perturbation with the wave number *q*, is relevant for the stability of the incoherent state. For given parameter sets, the incoherent state is stable if and only if the growth rates *Re*(λ_*q*_) for all possible *q* are non-positive. For convenience, we first treat *q* in Eq (10) as a real number. Fig 1 shows the growth rate *Re*(λ_*q*_) against real number *q* for several coupling strengths and coupling radius with other parameters fixed. With the increase of |*q*|, the growth rate *Re*(λ) displays a trend of damping oscillation whose period is determined by the joint parameter *σq*. Depending on the coupling strength, the maximum of the growth rate may occur at either *q*_*m*_ = 0 or a nonzero *K*-dependent *q*_*m*_. That is, the perturbation taking the form of the spatial modes with the wave numbers close to *q*_*m*_ are the most dangerous to the stability of the incoherent state. The right insets in Fig 1 identifying *q*_*m*_ against *K* show nonzero *q*_*m*_ for intermediate coupling strength *K* and zero *q*_*m*_ otherwise. It is interesting to note that the intermediate coupling strength occupies the range of (1, 2.5) and is insensitive to the coupling radius *σ*. In the range of intermediate coupling strength *K*, *q*_*m*_ increases with the coupling strength. As we have mentioned, the wave number *q* is treated as a real number in Eq (10) for the convenience of solving the equation. However, a periodic boundary condition with respect to *x* in the model (1) requires *q* to be integers, therefore, the right insets in Fig 1 show that the most dangerous spatial modes could be *q*_*m*_ = 0, 1 for *σ* = 0.4, *q*_*m*_ = 0, 1, 2 for *σ* = 0.2, while *q*_*m*_ ≤ 20 for *σ* = 0.02. In other words, decreasing the coupling radius leads that more spatial modes should be considered for the stability of the incoherent state. Besides, we present *Re*(λ_0_), *Re*(λ_1_) and *Re*(λ_2_) against *K* by the left insets in Fig 1, from which we can determine the stability of the corresponding partially coherent twisted states.

**Fig 2:**
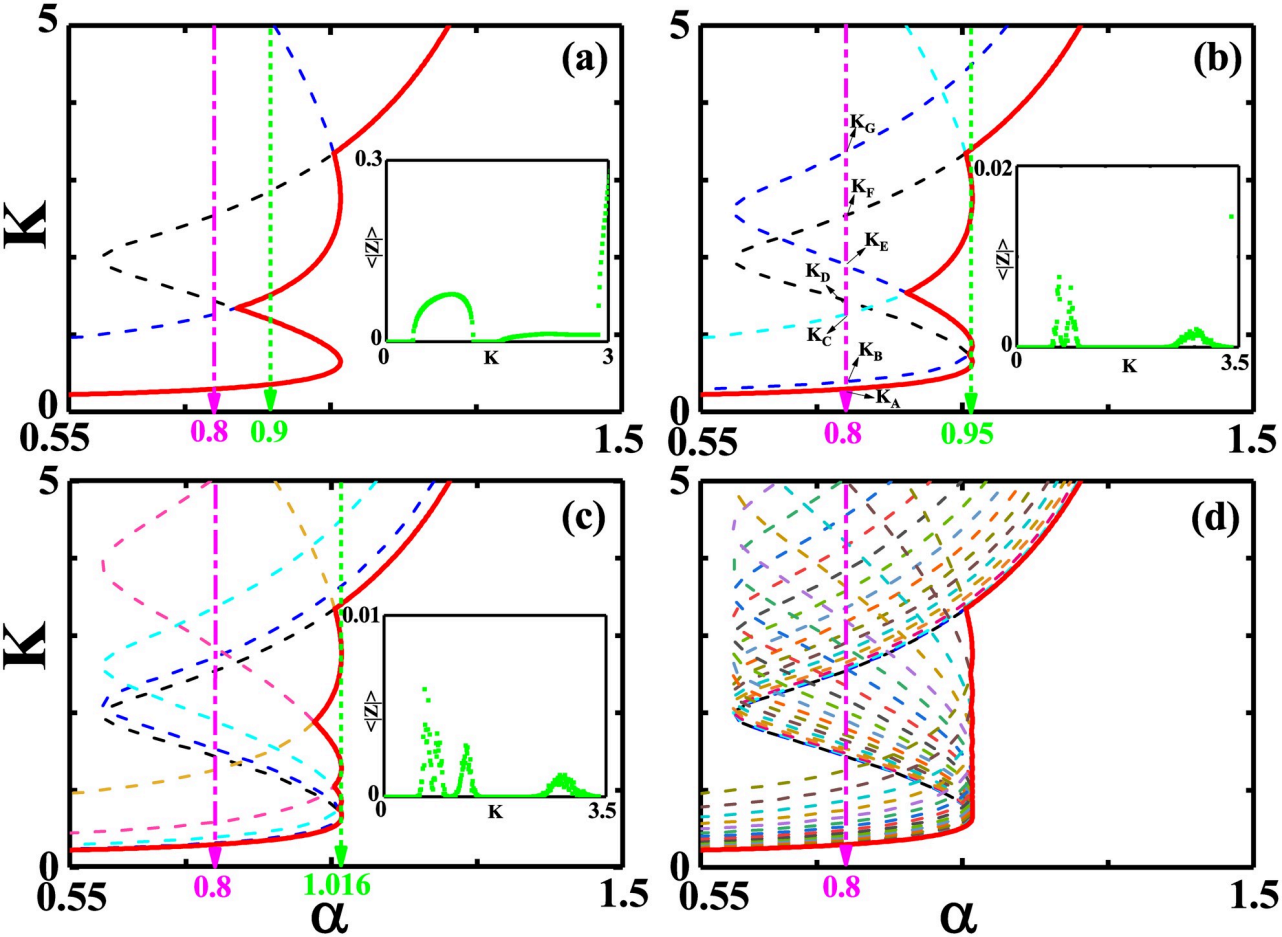
The stability diagrams of the incoherent state in the plane of the phase lag *α* and the coupling strength *K* for different coupling radius. In each plot, we present the critical curves with *Re*(λ_*q*_) = 0 for different *q* and the boundary of the stability regime of the incoherent state, denoted by the curve in red, is made up of these critical curves with the largest *α* at a given *K*. (a) *σ* = 0.4, and *q* = 0, 1. *q* = 0 in black and *q* = 1 in blue. (b) *σ* = 0.2, and *q* = 0, 1, 2. *q* = 0 in black, *q* = 1 in blue and *q* = 2 in cyan. (c) *σ* = 0.1, and *q* = 0, 1, ⋯, 4. (d) *σ* = 0.02, and *q* ≤ 20. Inset in each plot shows 〈|*Z*|〉 against *K* for the intermediate coupling strength at *α* denoted by the green line. *γ*_1_ = 1, *γ*_2_ = 0.01, and *p* = 0.9. The denoted coupling strengths in (b): *K*_*A*_ ≃ 0.29, *K*_*B*_ ≃ 0.39, *K*_*C*_ ≃ 1.27, *K*_*D*_ ≃ 1.43, *K*_*E*_ ≃ 1.9, *K*_*F*_ ≃ 2.5, and *K*_*G*_ = 3.6.

Then we present the stability diagram of the incoherent state in the plane of the phase lag *α* and the coupling strength *K*. We take *σ* = 0.4 as an example where the spatial modes *q* = 0 and *q* = 1 should be considered. The critical curves with the growth rates *Re*(λ_0_) = 0 and *Re*(λ_1_) = 0 are plotted in Fig 2(a) and the intersection of the parameter regimes on the right side of critical curves gives the stability regime of the incoherent state. To be addressed, the critical curve of *Re*(λ_0_), independent of the coupling radius *σ*, actually gives rise to the boundary of the stability regime for the incoherent state in globally coupled phase oscillators where *σ* = 0.5. The *S*-shaped critical curve *Re*(λ_0_) = 0 suggests that, in a certain range of *α*, there is another stability island for the incoherent state in the globally coupled oscillators besides the coupling strength *K* close to zero [4]. The resurgence of the incoherent state exists when *σ* = 0.4 (the nonlocally coupled oscillators), which is exemplified by the inset where 〈|*Z*|〉 against *K* at *α* = 0.9 suggests one stability island at intermediate coupling strength. To be noted, with the assistance of the spatial mode *q*_*m*_ = 1, there may be two stability islands for the incoherent state with the increase of *α* at *σ* = 0.4. With the decrease of *σ*, more spatial modes endangering the incoherent state have to be considered. Consequently, more stability islands for the incoherent state appear and the range of *α* supporting the non-universal synchronization transitions narrows [see Fig 2(b) and 2(c)]. In the limit *σ* → 0, the stability island of the incoherent state is lost and the forward critical coupling strength for synchronization transition undergoes a jump at a certain *α*, which is hinted in Fig 2(d) where *σ* = 0.02. It is worth mentioning that the stability islands of the incoherent state appear in the range of intermediate coupling strength where the most dangerous spatial mode has nonzero wave number.

### Partially coherent twisted state

**Fig 3:**
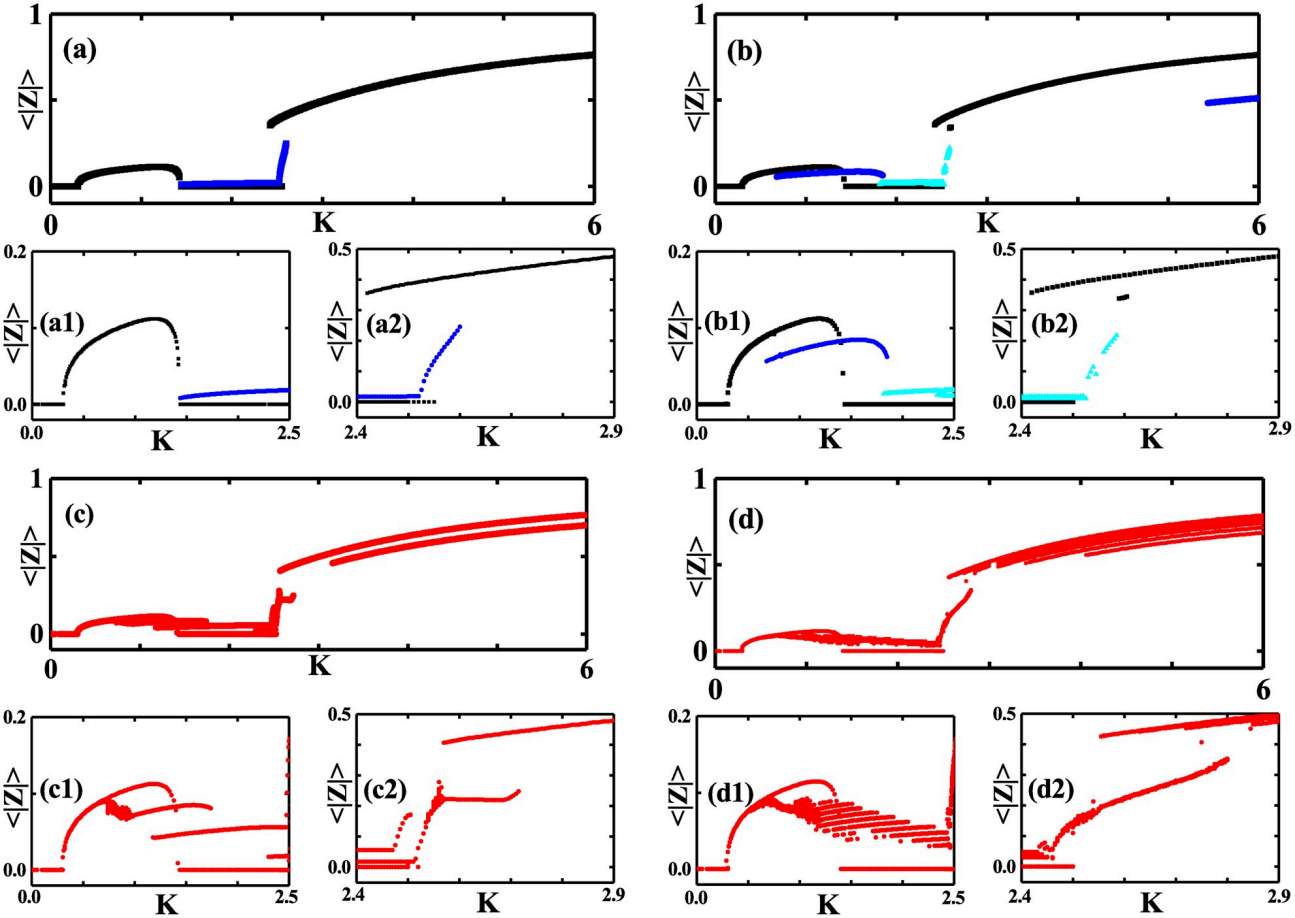
〈|*Z*|〉 against *K* for initial conditions taking the form of *u*_1,2_(*x*, *t*)∼*e*^*iqx*^ with different integers *q*. (a) *σ* = 0.4. *q* = 0 in black and *q* = 1 in blue. (b) *σ* = 0.2. *q* = 0 in black, *q* = 1 in blue and *q* = 2 in cyan. (c) *σ* = 0.1 and red for *q*. (d) *σ* = 0.02 and red for *q*. The magnifications for the range of *K* ∈ (0, 2.5) are presented in (a1-d1) and for the range of *K* ∈ (2.4, 2.9) in (a2-d2). *γ*_1_ = 1, *γ*_2_ = 0.01, *α* = 0.8, and *p* = 0.9.

When the incoherent state becomes unstable, the synchronous states pop up. We investigate the transition scenario from the incoherence to synchronous behaviors with the coupling strength *K* at a typical phase lag, for example *α* = 0.8, with other parameters the same as those in Fig 2. For each coupling strength, we consider several realizations, each with initial conditions taking the form of *u*_1,2_(*x*, *t*)∼*e*^*iqx*^ with a specific integer *q* and different ones with different *q*. That is, we use different initial conditions for each fixed *K*. Fig 3 presents 〈|*Z*|〉 against the coupling strength *K* for different coupling radius *σ* at *α* = 0.8 and, for better illustrations on the behavior of 〈|*Z*|〉, we zoom in two regions: *K* ∈ (0, 2.5) and *K* ∈ (2.4, 2.9). As shown in Fig 3, the threshold *K* (*K* = 0.29) beyond which synchronization steps in for the first time is independent of *σ*, which is in agreement with Fig 2 where the line *α* = 0.8 intersects with the critical curve *Re*(λ_0_) = 0 at the same *K*. 〈|*Z*|〉 behaves nonmonotonically with *K* and, especially, 〈|*Z*|〉 shows a valley in the range of the intermediate coupling strength. An important feature revealed in Fig 3 is the multistability. There exist several synchronous states for the intermediate and the strong coupling strength and the number of different states increases with the coupling radius decrease.

**Fig 4:**
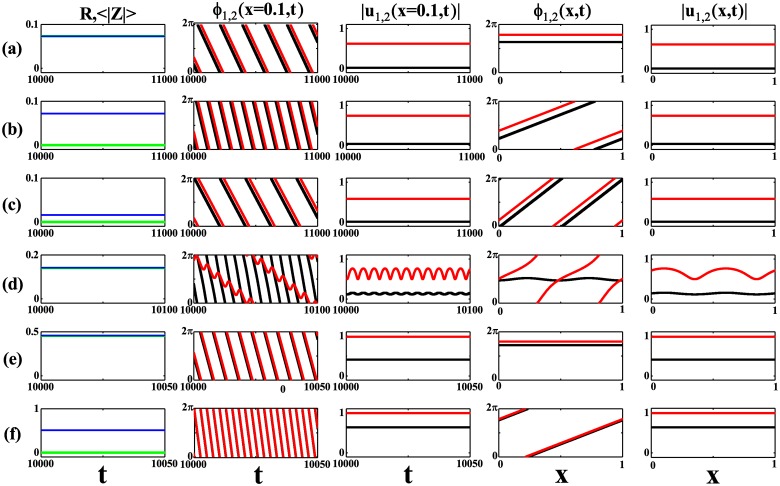
Typical twisted states at *σ* = 0.2. (a) The twisted state with *q* = 0 and |*u*_1_(*x*, *t*)| close to 0 at *K* = 0.5. (b) The twisted state with *q* = 1 and |*u*_1_(*x*, *t*)| close to 0 at *K* = 1. (c) The twisted state with *q* = 2 and |*u*_1_(*x*, *t*)| close to 0 at *K* = 2. (d) The modulated twisted state with *q* = 2 at *K* = 2.55. (e) The twisted state with *q* = 0 and |*u*_1_(*x*, *t*)| far away from 0 at *K* = 3. (f) The twisted state with *q* = 1 and |*u*_1_(*x*, *t*)| far away from 0 at *K* = 6. The first column shows the evolutions of the global quantities *R* (in green) and 〈|*Z*|〉 (in blue). The second column shows the evolutions of *ϕ*_1_(*x* = 0.1, *t*) (in black) and *ϕ*_2_(*x* = 0.1, *t*) (in red) and the third column shows |*u*_1_(*x* = 0.1, *t*)| (in black) and |*u*_2_(*x* = 0.1, *t*)| (in red). The fourth column shows the snapshots of *ϕ*_1_(*x*, *t*) (in black) and *ϕ*_2_(*x*, *t*) (in red) and the fifth column the snapshots of |*u*_1_(*x*, *t*)| (in black) and |*u*_2_(*x*, *t*)| (in red). *γ*_1_ = 1, *γ*_2_ = 0.01, *α* = 0.8, and *p* = 0.9.

To identify these different synchronous states and understand the dependence of 〈|*Z*|〉 on *K*, we take *σ* = 0.2 as an example. Following the line (shown by the dashed line *α* = 0.8 in magenta) in Fig 2(b) when *K* increases from zero, we encounter several critical coupling strengths, from *K*_*A*_ to *K*_*G*_, at which one of *Re*(λ_*q*_) becomes zero and signals the alternation between the stability and the instability of the incoherent state in response to the perturbation along the spatial modes *e*^*iqx*^. When *K* ∈ (*K*_*A*_, *K*_*D*_), *Re*(λ_0_) becomes positive and the incoherent state is unstable with respect to the homogeneous perturbation. As a result, in Fig 3(b1), 〈|*Z*|〉 rises from zero at *K*_*A*_ and falls to zero at *K*_*D*_. Fig 4(a) where *K* = 0.5 in the range of *K*_*A*_ to *K*_*D*_ shows a partially coherent twisted state with *q* = 0 characterized by *ϕ*_1,2_(*x*, *t*) = *ϕ*_1,2_(*t*), a homogeneous time-periodic argument, and |*u*_1,2_(*x*, *t*)| = |*u*_1,2_|, an amplitude independent of time and space. In this twisted state, |*u*_2_| ≫ |*u*_1_| with |*u*_1_| close to zero and two global quantities, *R* and 〈|*Z*|〉, are almost the same. When *K* ∈ (*K*_*B*_, *K*_*E*_), *Re*(λ_1_) becomes positive, which gives rise to a twisted state with *q* = 1. In this range, 〈|*Z*|〉 becomes nonzero in response to the perturbation taking the form of *e*^*ix*^. Fig 4(b) shows a twisted state with *q* = 1 at *K* = 1 where *ϕ*_1,2_(*x*, *t*) varies 2*π* along the ring and |*u*_1,2_(*x*, *t*)| = |*u*_1,2_|. In this *q* = 1 twisted state, *R* ≃ 0 due to the fact that the contributions from the locations *x* and *x* + *L*/2 are cancelled out since *e*^*iϕ*_1,2_(*x*, *t*)^ + *e*^*iϕ*_1,2_(*x* + *L*/2, *t*)^ = 0. Furthermore, *Re*(λ_2_) becomes positive from negative at *K*_*C*_, which may be confirmed in Fig 3(b1), and induces a twisted state with *q* = 2 presented in Fig 4(c) where *ϕ*_1,2_ change 4*π* along the ring. *Re*(λ_1_) becomes positive again when the coupling strength is increased to *K* = *K*_*G*_. However, the new *q* = 1 twisted state [see Fig 4(f)] pops up till *K* ≃ 5.4. It is because that the new *q* = 1 twisted state may exist since *K* = *K*_*G*_ but it becomes stable only till a larger *K*, which can be proved by the stability analysis of the twisted state following Ref. [15]. To be stressed, *Re*(λ_2_)>0 is kept even when *K* = 5. However, the twisted state with *q* = 2 may undergo a secondary bifurcation and leads to a modulated twisted state. In the modulated twisted state, as shown in Fig 4(d) where *K* = 2.55, |*u*_1,2_| become space-dependent with *ϕ*_2_ varying 4*π* along the ring and *ϕ*_1_ undergoing a small amplitude oscillation with spatial period *L*/2. More interestingly, |*u*_1,2_(*x*, *t*)| become periodic in time in this modulated twisted state. Actually, the bifurcation to the modulated twisted state with *q* = 2 occurs at *K* ≃ 2.52 and Fig 3(b2) suggests a continuous transition between the time-independent and the modulated twisted states with *q* = 2. When increasing *K* to a strong coupling strength *K*_*F*_, *Re*(λ_0_) becomes positive again, which leads to another twisted state with *q* = 0. As shown in Fig 4(e) with *K* = 3, this new twisted state with *q* = 0 is characterized by *ϕ*_1,2_(*x*, *t*) = *ϕ*_1,2_(*t*) and |*u*_1,2_(*x*, *t*)| = |*u*_1,2_|. The new state is quite different from the *q* = 0 state in Fig 4(a) in the sense that, here, both |*u*_1_| and |*u*_2_| are far away from 0. Due to the frequency distribution Eq (2), we may partition phase oscillators into two groups, one with the Lorentzian distribution with half width *γ*_1_ and the other with the Lorentzian distribution with half width *γ*_2_. Consider that *u*_1,2_(*x*, *t*) = *a*(−*iγ*_1,2_, *x*, *t*) play the order parameters measuring the coherence in groups of oscillators with the half widths *γ*_1,2_ in the frequency distributions, the difference between these two twisted states can be apprehended as following. In the twisted state with *q* = 0 in Fig 4(a), |*u*_1_(*x*)≃0| suggests that only oscillators in *γ*_2_ group get partially synchronized while those in the *γ*_1_ group are desynchronized. In contrast, in the twisted state with *q* = 0 in Fig 4(e), |*u*_1,2_| far away from zero indicate that oscillators in both the *γ*_2_ group and the *γ*_1_ group get partially synchronized. The similar cause results in the two different twisted states for *q* = 1. Noting that |*u*_1,2_| are far away from zero in Fig 4(f), we know that oscillators in both the *γ*_1_ and the *γ*_2_ groups are in partially synchronous state, which is different from the twisted state with *q* = 1 in Fig 4(b) where oscillators in the *γ*_1_ group are asynchronous.

Accordingly, the transition scenarios in Fig 3(a), 3(c) and 3(d) can be analyzed in the same way. Briefly, the synchronous states presented in Figs 3 and 4 originate from the instabilities of the incoherent state to the perturbations in different spatial modes. Based on Fig 2, the multistability in Eqs (1) and (8) may be observed when there exist several spatial modes with positive *Re*(λ_*q*_) at the same parameter set, and it could be understood that decreasing *σ* leads to more and more synchronous states. In addition, it has to be addressed that there are two types of twisted states with the same winding number *q*, one with *u*_1_ ≃ 0 in weak and intermediate coupling strength and the other with *u*_1_ far away from 0 in strong coupling strength. In the former one, only oscillators in the *γ*_2_ group get partially synchronized while, in the latter one, oscillators from both *γ*_1_ and *γ*_2_ groups get partially synchronized.

### Complicated spatiotemporal patterns

**Fig 5:**
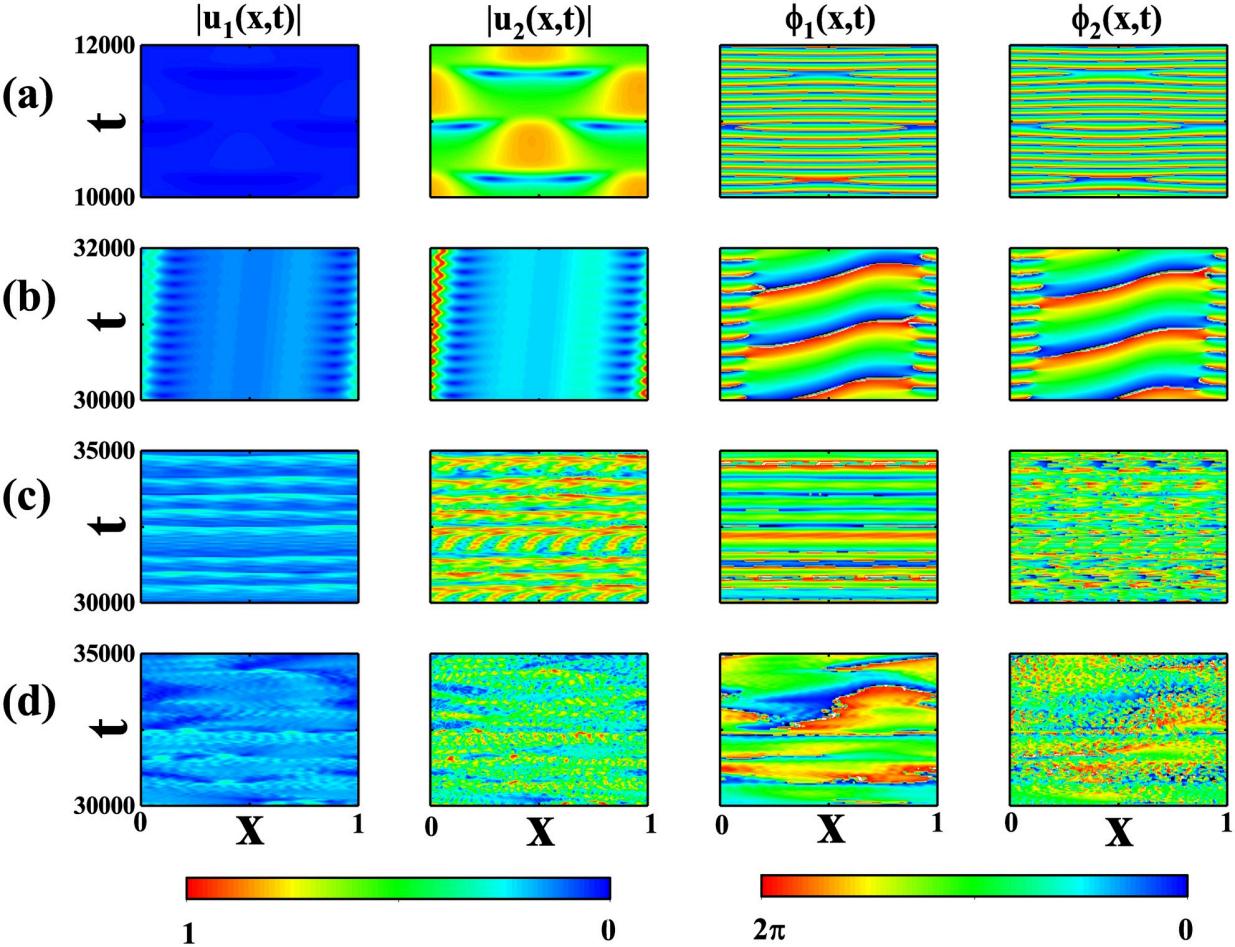
Several complicated spatialtemporal patterns. (a) The standing-wave-like pattern at *σ* = 0.2 and *K* = 0.8. (b) The soliton-like pattern at *σ* = 0.02 and *K* = 2, 52. (c) Weak spatialtemporal chaos at *σ* = 0.08 and *K* = 2.52. (d) The spatialtemporal chaos at *σ* = 0.02 and *K* = 2.52. The first column shows the evolution of |*u*_1_(*x*, *t*)|, the second column shows the evolution of |*u*_2_(*x*, *t*)|, the third column shows the evolution of *ϕ*_1_(*x*, *t*), and the fourth column shows the evolution of *ϕ*_2_(*x*, *t*). *γ*_1_ = 1, *γ*_2_ = 0.01, *α* = 0.8, and *p* = 0.9.

We have presented several twisted states in correspondence to the response of the incoherent states to perturbation on different spatial modes. However, there are some parameter regions in which there are several different spatial modes unstable to the incoherent state. If we consider arbitrary initial conditions, the competition among different spatial modes may lead to complicated spatiotemporal patterns. Here, we present some of them. Fig 5(a) shows a periodic spatiotemporal pattern. The amplitudes |*u*_1,2_(*x*, *t*)| display characteristics of standing wave state with spatial periodicity *L* while *ϕ*_1,2_(*x*, *t*) perform periodic oscillation in the presence of some phase slips. Fig 5(b) shows an interesting spatialtemporal patterns. In the respect of |*u*_1,2_(*x*, *t*)|, the pattern looks like a localized structure traveling along the ring. At the same time, the space is divided into two moving regions in the respect of *ϕ*_1,2_(*x*, *t*) and, in different regions, *ϕ*_1,2_(*x*, *t*) increases at different velocities. The model also displays spatialtemporal chaos. Fig 5(c) shows a weak spatialtemporal chaos in which |*u*_1,2_(*x*, *t*)| display a little regularity while Fig 5(d) shows a more turbulent state for both |*u*_1,2_(*x*, *t*)| and *ϕ*_1,2_(*x*, *t*). According to the classification in a recent work [20], the states in Fig 5(c) and 5(d) belong to the amplitude turbulence since |*u*_1,2_(*x*, *t*)| = 0 may be encountered at any spatial location.

## Conclusion

In summary, we studied a ring of nonlocally coupled phase oscillators in which the frequency distribution is made up of two Lorentzians with the same center frequency but with different half widths. Using OA ansatz, we derived a reduced model in the limit of infinite number of oscillators. Based on the reduced model, we studied analytically the stability of the incoherent state and found that the most unstable spatial mode to the incoherent state depends on the coupling strength, the homogeneous perturbation with the winding number *q*_*m*_ = 0 for the weak and the strong coupling strength and inhomogeneous perturbation with nonzero winding number *q*_*m*_ ≠ 0 for intermediate coupling strength. The critical curves of the different spatial modes to the incoherent state may be *S*-shaped ones, which may give rise to multiple stability islands of the incoherent state. By numerically simulating the reduced equations, we found a large number of twisted states which result from the response of the incoherent state to the perturbations in the form of different spatial modes. Especially, there are two types of twisted states, one for the intermediate coupling strength and the other for the strong coupling strength. By partitioning oscillators into two groups each of which obeys a unimodal frequency distribution, we found that, for the twisted state for the intermediate coupling strength, the group of oscillators with fat frequency distribution remains in desynchronization and, for the states for strong coupling strength, both groups are in partial synchronization.
